# Effectiveness of Blended Versus Traditional Refresher Training for Cardiopulmonary Resuscitation: Prospective Observational Study

**DOI:** 10.2196/52230

**Published:** 2024-04-29

**Authors:** Cheng-Yu Chien, Shang-Li Tsai, Chien-Hsiung Huang, Ming-Fang Wang, Chi-Chun Lin, Chen-Bin Chen, Li-Heng Tsai, Hsiao-Jung Tseng, Yan-Bo Huang, Chip-Jin Ng

**Affiliations:** 1 Department of Emergency Medicine Chang Gung Memorial Hospital Linkou Branch Taoyuan Taiwan; 2 Department of Emergency Medicine Chang Gung Memorial Hospital Taipei Branch Taipei Taiwan; 3 Department of Emergency Medicine Ton-Yen General Hospital Zhubei Taiwan; 4 Institute of Epidemiology and Preventive Medicine College of Public Health, National Taiwan University Taipei Taiwan; 5 Department of Senior Service Industry Management Minghsin University of Science and Technology Hsinchu Taiwan; 6 Graduate Institute of Management College of Management, Chang Gung University Taoyuan Taiwan; 7 Department of Emergency Medicine New Taipei City Hospital New Taipei City Taiwan; 8 Department of Emergency Medicine New Taipei Municipal TuCheng Hospital and Chang Gung University New Taipei Taiwan; 9 Department of Nursing Chang Gung University of Science and Technology Taoyuan Taiwan

**Keywords:** cardiopulmonary resuscitation, blended method, blended, hybrid, refresher, refreshers, teaching, instruction, observational, training, professional development, continuing education, retraining, traditional method, self-directed learning, resuscitation, CPR, emergency, resuscitation, rescue, life support, cardiac, cardiopulmonary

## Abstract

**Background:**

Generally, cardiopulmonary resuscitation (CPR) skills decline substantially over time. By combining web-based self-regulated learning with hands-on practice, blended training can be a time- and resource-efficient approach enabling individuals to acquire or refresh CPR skills at their convenience. However, few studies have evaluated the effectiveness of blended CPR refresher training compared with that of the traditional method.

**Objective:**

This study investigated and compared the effectiveness of traditional and blended CPR training through 6-month and 12-month refresher sessions with CPR ability indicators.

**Methods:**

This study recruited participants aged ≥18 years from the Automated External Defibrillator Donation Project. The participants were divided into 4 groups based on the format of the CPR training and refresher training received: (1) initial traditional training (a 30-minute instructor-led, hands-on session) and 6-month traditional refresher training (Traditional6 group), (2) initial traditional training and 6-month blended refresher training (an 18-minute e-learning module; Mixed6 group), (3) initial traditional training and 12-month blended refresher training (Mixed12 group), and (4) initial blended training and 6-month blended refresher training (Blended6 group). CPR knowledge and performance were evaluated immediately after initial training. For each group, following initial training but before refresher training, a learning effectiveness assessment was conducted at 12 and 24 months. CPR knowledge was assessed using a written test with 15 multiple-choice questions, and CPR performance was assessed through an examiner-rated skill test and objectively through manikin feedback. A generalized estimating equation model was used to analyze changes in CPR ability indicators.

**Results:**

This study recruited 1163 participants (mean age 41.82, SD 11.6 years; n=725, 62.3% female), with 332 (28.5%), 270 (23.2%), 258 (22.2%), and 303 (26.1%) participants in the Mixed6, Traditional6, Mixed12, and Blended6 groups, respectively. No significant between-group difference was observed in knowledge acquisition after initial training (*P*=.23). All groups met the criteria for high-quality CPR skills (ie, average compression depth: 5-6 cm; average compression rate: 100-120 beats/min; chest recoil rate: >80%); however, a higher proportion (98/303, 32.3%) of participants receiving blended training initially demonstrated high-quality CPR skills. At 12 and 24 months, CPR skills had declined in all the groups, but the decline was significantly higher in the Mixed12 group, whereas the differences were not significant between the other groups. This finding indicates that frequent retraining can maintain high-quality CPR skills and that blended refresher training is as effective as traditional refresher training.

**Conclusions:**

Our findings indicate that 6-month refresher training sessions for CPR are more effective for maintaining high-quality CPR skills, and that as refreshers, self-learning e-modules are as effective as instructor-led sessions. Although the blended learning approach is cost and resource effective, factors such as participant demographics, training environment, and level of engagement must be considered to maximize the potential of this approach.

**Trial Registration:**

IGOGO NCT05659108; https://www.cgmh-igogo.tw

## Introduction

Sudden cardiac arrest is a severe condition, particularly when it occurs outside a medical facility, and the corresponding survival rates are very low. In Europe and North America, these survival rates range from 7% to 13%, whereas in Asia, they are even lower at 0.5% to 8.5% [1-3]. Furthermore, these survival rates vary significantly by location and demography. Some countries exhibit higher survival rates, ranging from 20% to 40%. In contrast, according to a database, the survival rate in Taiwan is 8% to 10% [3-6]. Therefore, survival after out-of-hospital cardiac arrest (OHCA) exhibits substantial variability across regions [7].

The survival status for OHCA is closely linked to the Chain of Survival of the American Heart Association (AHA), which emphasizes the early activation of emergency medical services (EMSs), early cardiopulmonary resuscitation (CPR), and early defibrillation as the first 3 critical links [8]. These 3 interventions can be administered in a prehospital setting, and achieving high-quality outcomes following these interventions is pivotal to enhancing OHCA survival rates. Owing to significant disparities in EMSs, bystander CPR rates, and public access to automated external defibrillators (AEDs) in different regions, OHCA survival rates exhibit corresponding variations [7]. However, through CPR training and dispatcher-assisted CPR, the global bystander CPR rate has improved from approximately 20% in 2001 to 40% to 55% in 2023 [9-11]. In Taiwan, the government has implemented legally mandated continuous public CPR education and training programs aimed at improving the response of bystanders to sudden cardiac arrest [12]. This effort has resulted in significant increases in bystander CPR rates and the use of public AEDs [7,13]. Over a decade, 14% and 3.8% increases have been noted in the bystander CPR rate and the use rate of public AEDs, respectively [6,9,14].

Research has demonstrated a significant decline in CPR skills over time, especially regarding chest compression depth and rate [15]. Consequently, maintaining the public’s CPR skills and their motivation for learning CPR is challenging. In response to this challenge, the AHA recommended self-directed training for CPR during the COVID-19 pandemic [16]. Similarly, the European Resuscitation Council recognized blended training models as an alternative to traditional face-to-face teaching models [17,18]. Furthermore, previous studies have indicated that blended training is not inferior to traditional methods and offers advantages such as resource saving and time saving, making it an effective approach for CPR education [15]. By using blended training models, which combine web-based self-guided learning with hands-on practice, individuals can acquire or refresh their CPR skills at their own pace and convenience [15]. Such flexibility fosters increased levels of engagement and enhanced retention of CPR knowledge and thus ultimately enhances the public’s preparedness for treating sudden cardiac arrests. Therefore, blended approaches are valuable both during a pandemic and when in-person training cannot be conducted, ensuring widespread CPR education for a broad audience [19].

Limited research has been conducted regarding the effective implementation of relearning stimuli to maintain CPR skills within the framework of blended training. Therefore, the primary objective of this study was to provide relearning stimuli in a blended training setting after using both traditional and blended teaching methods; this study also investigated the effectiveness and most appropriate frequency of blended training. Finally, this study compared learners’ performance in 2 educational settings. We hypothesized that using the blended method with 6-month interventions would yield outcomes comparable to those achieved through the traditional method.

## Methods

### Study Design, Setting, and Participants

**Figure 1:**
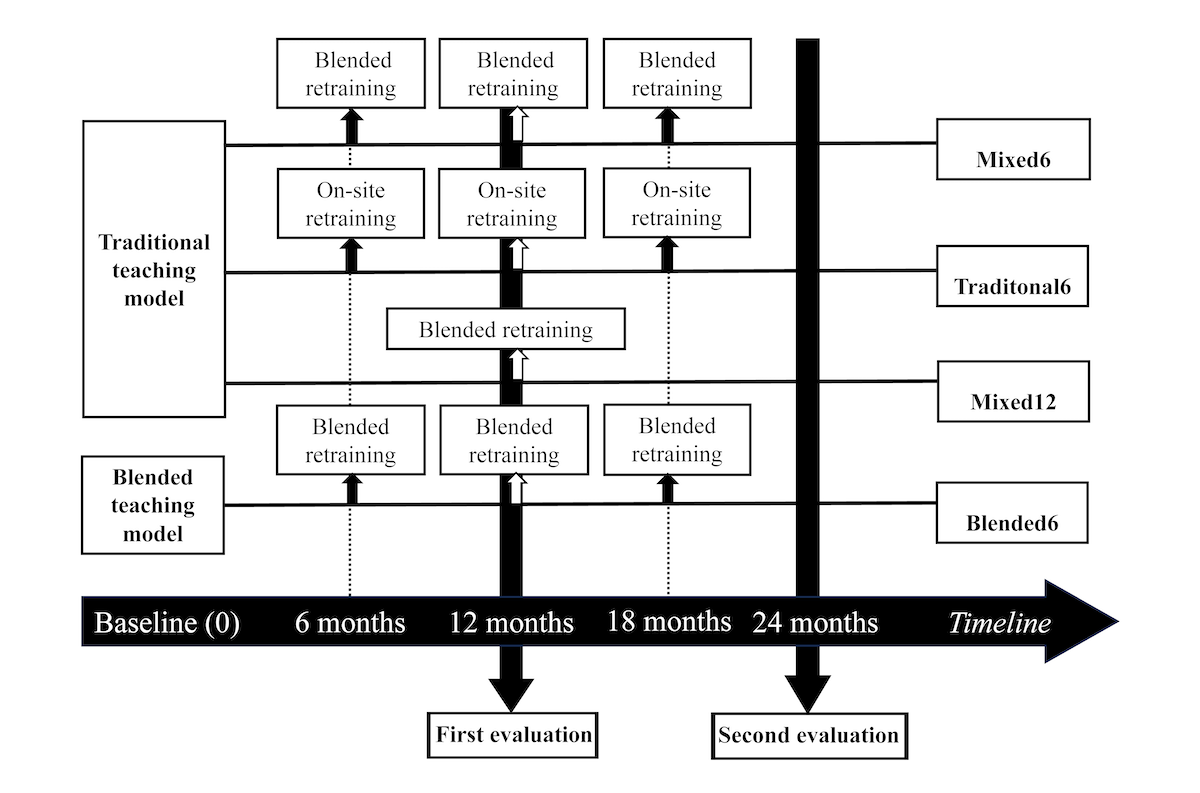
Schematic diagram illustrating the arrangement of four training courses: Mixed6, Traditional6, Mixed12 and Blended6.

This study used a prospective observational design, and participants were recruited from the AED Donation Project, also called the Love GOGO program, implemented by Chang Gung Memorial Hospital, Taiwan. The Love GOGO program aims to establish an educational training system for CPR and build a comprehensive teaching database encompassing participants’ attributes, learning models, and CPR parameters. Individuals from government agencies, nonprofit organizations, schools, and organizations required by current Taiwanese regulations to have AED facilities participate in this education and training program. These include transportation hubs, large long-distance vehicles, tourist spots, schools or large assembly places, large leisure places, large shopping malls, hotels, large public bathhouses, hot springs, and public service sectors such as police stations. These organizations voluntarily participated in the Love GOGO program and proactively contacted the research assistant (YTK) of this study. For this study, participants were enrolled in the Love GOGO program from January to December 2017. Based on our previous study, both traditional and blended teaching models showed a noticeable decline in skill retention after approximately 6 months [12,15]. In this study, mandatory retraining was administered every 6 months or 1 year (Figure 1), spanning a comprehensive training regimen conducted over 2 years. In the initial training phase, the participants were assigned to either traditional teaching or blended teaching modes. Learning effectiveness assessments were conducted every 12 months, with a retraining frequency of 6 or 12 months. Before refresher courses but following initial training, each group underwent evaluation at 12 and 24 months. The results of the 12-month learning effectiveness assessment were disclosed only at 24 months. The research assistants independently allocated training methodologies and the frequencies of subsequent follow-up assessments, using unit convenience and considering the practicalities of the study context. Those responsible for the execution of course training and assessments were not involved in the allocation process.

The inclusion criteria are described as follows: (1) aged at least 18 years and (2) not having undergone any CPR training within the preceding 2 years. Individuals who had physical limitations preventing them from kneeling to perform CPR, who were pregnant, or who were unwilling to sign the informed consent form were excluded from this study. Before initial training, the research assistant divided the participants into groups, and their basic characteristics—namely age, sex, educational level, exercise habits, whether they were receiving CPR training for the first time, their most recent CPR training, and their basic life support (BLS) knowledge scores—were collected through a web-based survey. The assessment of CPR learning should encompass the status of both knowledge and skills. After initial training but before refresher training, we collected data regarding BLS knowledge, skill tests, and CPR quality at the scene at 12 and 24 months. The BLS knowledge and skill tests received approval from the Chairman of the Taiwan Society of Emergency Medicine and have also been published in previous studies [12,15] (Multimedia Appendices 1 and 2).

### Ethical Considerations

**Figure 2:**
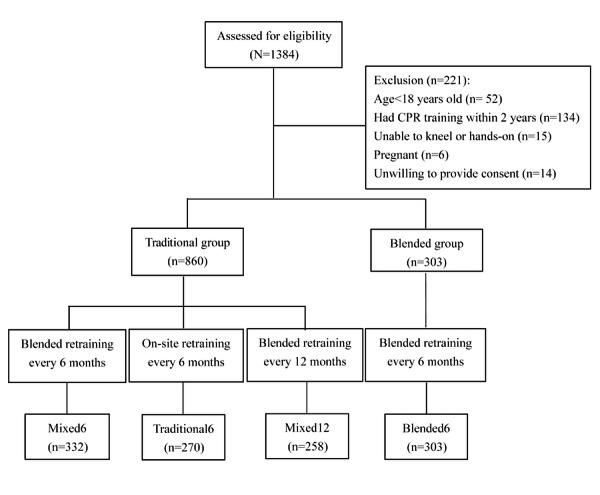
Flow diagram of participants’ inclusion and allocation. CPR: cardiopulmonary resuscitation.

This study was approved by the institutional review board of the Chang Gung Memorial Foundation (approvals: 201600149B0, 201900399B0, 202200559B0, CMRPG1M0081, and CMRPG1N0081), and this study was performed in accordance with relevant guidelines and regulatory requirements. The IGOGO database is anonymized or deidentified, and no type of compensation is provided to participants. Written informed consent was obtained from all the participants (Figure 2).

### Sample Size

An appropriate sample size for this study was estimated based on a pilot study, in which the expected percentage of correct compression depth was 65.4 (SD 29.5) cm for traditional training. To achieve a statistical power of 90% by using a 2-tailed *t* test with a significance level of *P*<.05, each group was required to have 225 participants. We planned to enroll at least 900 participants in total.

### Interventions

The Love GOGO program offers 2 teaching models for CPR training: the traditional instructor-led, classroom-based model and the blended model. In the traditional model, participants undergo a 90-minute session, which includes a 60-minute CPR knowledge education session involving a CPR lecture and demonstration, an AED use demonstration, an introduction to relevant laws, and a 30-minute hands-on practice session focusing on compression-only CPR. The blended program, which was approved by the Chairman of the Taiwan Society of Emergency Medicine in 2016, combines an 18-minute e-learning module with a 30-minute hands-on session for compression-only CPR. The e-learning module comprises a video that covers essential knowledge related to CPR and AEDs, including knowledge related to cardiac arrest scenes, the technique of compression-only CPR, the benefits of using CPR and AEDs for OHCA treatment, CPR and AED use steps, and an introduction to relevant laws. In this study, the participants assigned to the blended program were granted access to the e-learning video 3 days before the hands-on session. After completing the e-learning module, the participants practiced their skills in a 30-minute instructor-led, hands-on session in a classroom setting. Both CPR training programs were conducted by AHA instructors who were also emergency physicians. For hands-on CPR practice, both groups used sensor-equipped manikins (Resusci Anne with QCPR, Laerdal Medical AS). The participant-to-manikin-to-instructor ratio per class was 6:3:1, involving 4 instructors and 6 examiners. The study team provided different certification learning stimuli (traditional and blended learning) at 2 frequencies: every 6 months (at 6, 12, 18, and 24 months) and every 12 months (at 12 and 24 months). To establish groups with unique frequencies, the research assistant (YTK) conducted allocation during the initial training phase. Therefore, the traditional teaching model was applied for initial training, and certification sessions for retraining occurring every 6 or 12 months were conducted using either the blended retraining model (18-minute e-learning module with self–hands-on practice for compression-only CPR) or the on-site retraining model (30-minute instructor-led, hands-on session). These groups were called Mixed6 (initial traditional training and 6-month blended refresher training), Traditional6 (initial traditional training and 6-month traditional refresher training), and Mixed12 (initial traditional training and 12-month blended refresher training). For the Blended6 group, initial training was conducted using the blended teaching model, and for certification stimuli every 6 months, the blended retraining model was applied (Figure 1).

### Outcome Measures

This study systematically assessed the participants’ CPR knowledge and performance at multiple time points. Initially, the CPR knowledge and performance of the participants were assessed immediately after training. Following initial training but before refresher training, subsequent evaluations of knowledge and performance were conducted at 12 and 24 months. CPR knowledge was examined through a written test comprising 15 multiple choice questions, with a maximum total score of 100. CPR performance was assessed through 2 methods: examiner-rated assessment and manikin feedback. Individual examiners meticulously assessed the participants’ ability to execute the BLS sequence, encompassing tasks from verifying scene safety to using an AED, with a maximum total score of 40. Objective assessment data regarding CPR quality—including compression depth, compression rate, and full chest recoil—were collected from manikin feedback. The assessment adhered to the 2015 AHA guidelines update for CPR and emergency cardiovascular care; high-quality CPR was characterized by the following three criteria: (1) achieving a compression depth of 5-6 cm, (2) maintaining a compression rate of 100-120 beats per minute (bpm), and (3) facilitating complete chest wall recoil of >80%. Notably, because of the focus on compression-only CPR, ventilation was excluded because it was therefore beyond the scope of the assessment in this study. The primary outcome measure was the comparison of high-quality CPR among the 4 groups. Secondary outcome measures were differences in the percentage of full chest recoil, the percentage of compressions delivered with adequate depth (5-6 cm), the percentage of compressions delivered at an adequate rate (100-120 bpm), written test scores, and examiner-rated skill test scores.

### Statistical Methods

Descriptive statistics are expressed as mean (SD) for continuous variables and as counts and percentages for categorical variables. Linear regression analysis was conducted to determine any differences in the mean values of baseline characteristics among the groups, with adjustment for control variables—namely age, sex, educational level, exercise habits, whether CPR training was being received for the first time, most recent CPR training, and pretest BLS knowledge scores, which were based on the significance test result and which were proposed in previous research [12,15,20]. After allocation, differences in characteristics among groups were observed. To mitigate potential biases introduced by this allocation method, we applied multiple linear regression analyses and generalized estimating equation (GEE) to adjust for these variations when evaluating outcomes (Multimedia Appendices 3 and 4). The chi-square test was used to assess the differences in proportions among the groups, and the general linear model, such as analysis of covariance, was used to test differences among the groups. The control variables—namely age, sex, educational level, exercise habits, whether CPR training was being received for the first time, and pretest BLS knowledge scores—may have influenced skill retention and test scores. Therefore, the model was adjusted for these variables.

We conducted the assessments of the participant’s skill levels and BLS knowledge scores at multiple time points. Accordingly, we used a GEE to examine changes over time in CPR ability indicators among the groups. This allows us to comprehend the changes in CPR skills among trainees under different training methods, using a GEE model to analyze the change over time in CPR ability indicators among groups. The GEE analysis was adjusted for the control variables. To ensure fairness, statistical analysis was conducted using data obtained at time points specific to each group. That is, only data from the postinitial training (baseline), 12-month, and 24-month assessments were included in the analysis.

CPR performance is displayed by line charts, bar charts, and radar charts. In particular, we generated radar charts to illustrate the relative CPR performance in each session. The scores were converted using percent ranking, and the average score was then calculated to represent the performance of each skill for each training method. Statistical analysis was conducted using SPSS Statistics (version 26.0; IBM Corp) and STATA (MP 16.0; Stata Corp LLC).

## Results

### Baseline Characteristics

A total of 1163 participants were recruited for this study, and they were allocated to 4 training groups. The mean age of the participants was 41.82 (SD 11.6) years, and 62.3% (n=725) of participants were female. In this study, 332 (28.5%), 270 (23.2%), 258 (22.2%), and 303 (26.1%) participants were placed in the Mixed6, Traditional6, Mixed12, and Blended6 groups, respectively. Table 1 displays the baseline characteristics of these 4 training groups. As this study was observational rather than randomized, significant differences were observed among the 4 training groups in terms of age (*P*<.001), sex (*P*=.008), educational level (*P*=.006), and CPR training experience (*P*<.001; Table 1). Notably, the Traditional6 group had the highest average age (45.30, SD 11.39 years) and consisted of 68.9% (186/270) female participants. Additionally, this group had the highest proportion of individuals receiving CPR training for the first time (92/270, 34.1%). However, no statistically significant difference was observed in the BLS pretest knowledge score (*P*=.11), with an overall mean score of 67.96 (SD 15.08); this finding indicated similar baseline performance across the groups before BLS training.

**Table 1:** Baseline characteristics of the 4 training groups.

Variables			Mixed6 (n=332)		Traditional6 (n=270)		Mixed12 (n=258)		Blended6 (n=303)		*P* value	
Age (years), mean (SD)			40.78 (9.97)		45.30 (11.39)		40.72 (12.34)		40.78 (12.28)		*<.001^a^*	
**Sex, n (%)**												*.008*
	Male	117 (35.2)		84 (31.1)		104 (40.3)		133 (43.9)				
	Female	215 (64.8)		186 (68.9)		154 (59.7)		170 (56.1)				
**Education, n (%)**												*.006*
	Below high school	2 (0.6)		26 (9.6)		6 (2.3)		23 (7.6)				
	High school, college education, and above	330 (99.4)		244 (90.4)		252 (97.7)		280 (92.4)				
Exercise habits, n (%)			142 (42.8)		116 (45.5)		123 (48.6)		120 (41.5)		.35	
First time for CPR^b^ training, n (%)			33 (9.9)		92 (34.1)		34 (13.2)		92 (30.4)		*<.00^a^*	
**Last CPR training, n (%)**												*<.001*
	Within 2-3 years	122 (36.7)		62 (23)		138 (53.5)		73 (24.1)				
	Over 3 years	181 (54.5)		196 (72.6)		109 (42.3)		205 (67.7)				
	Not clear	29 (8.8)		12 (4.4)		11 (4.2)		25 (8.2)				
BLS pretest knowledge score^b^, mean (SD)			67.78 (13.15)		67.96 (15.08)		70.57 (15.97)		68.17 (16.12)		.11	

^a^Italic formatting indicates that there is a statistically significant difference in the *P* value.

^b^CPR: cardiopulmonary resuscitation.

^c^BLS: basic life support.

### Posttraining Assessment

According to the results of the objective assessment after the first training session, significant differences were found among the 4 groups in skill tests (*P*=.002), average chest compression depth (*P*<.001), and average compression rate (*P*<.001; Table 2) after adjustment for the control variables in the multivariate analysis (Multimedia Appendix 5). In the multivariate analysis, higher skill test scores were associated with younger age (*P*=.003), higher educational level (*P*<.001), more previous CPR training experience (*P*=.04), and higher BLS pretest scores (*P*=.004). Furthermore, the average compression depth was significantly associated with age (*P*=.02) and sex (*P*<.001), and the average compression rate was significantly associated with educational level (*P*=.04) and CPR training experience (*P*=.02). Although the mean chest compression depths differed among the 4 groups, the proportion of participants achieving the correct chest compression depth did not differ on average (*P*=.11). For the overall performance assessment, the proportion of participants achieving high-quality CPR ranged from 27.4% (91/332) to 32.3% (98/303). The lowest proportion was observed in the Mixed6 group, and the highest proportion was found in the Blended6 group. In the multivariate analysis, high-quality CPR was negatively correlated with the Mixed12 training method (adjusted odds ratio 0.65, 95% CI 0.45-0.93; *P*=.02; Multimedia Appendix 6).

**Table 2:** Postinitial training evaluation (baseline) for the 4 training groups.

Variables	Mixed6 (n=332)	Traditional6 (n=270)	Mixed12 (n=258)	Blended6 (n=303)	*P* value^a^
BLS^b^ knowledge score, mean (SD)	86.05 (11.38)	84.61 (12.96)	86.76 (11.79)	84.10 (11.19)	.23
Skill test, mean (SD)	35.09 (3.26)	35.81 (2.78)	35.73 (3.76)	35.26 (4.05)	*.002^c^*
Average chest compression depth (cm), mean (SD)	5.07 (0.74)	5.01 (0.73)	5.23 (0.43)	5.33 (0.57)	*<.001*
Average chest compression rate (times per minute), mean (SD)	113.88 (13.87)	110.56 (14.34)	116.07 (11.33)	116.65 (10.28)	*<.001*
Correct compression depth, mean (SD)	70.79 (32.83)	71.24 (30.55)	74.75 (32.21)	75.88 (33.31)	.11
Correct compression rate, mean (SD)	61.14 (31.87)	66.16 (30.57)	68.61 (34.15)	61.98 (34.94)	*.01*
Correct recoil, mean (SD)	84.39 (35.29)	87.16 (30.32)	79.72 (37.57)	80.35 (35.65)	.20
High-quality CPR^d,e^, n (%)	91 (27.4)	86 (31.8)	77 (29.8)	98 (32.3)	.52

^a^The *P* value was obtained from the general linear regression model adjusted for age, sex, educational level, exercise habits, whether CPR training was being received for the first time, and BLS pretest knowledge score.

^b^BLS: basic life support.

^c^Italic formatting indicates that there is a statistically significant difference in the *P* value.

^d^CPR: cardiopulmonary resuscitation.

^e^*P* values obtained from the chi-square test. High-quality CPR was denoted by an average compression depth between 5 and 6 cm, an average compression rate of 100-120 beats per minute, and 80% chest recoil.

### Posttraining Follow-Up and Maintenance

**Figure 3:**
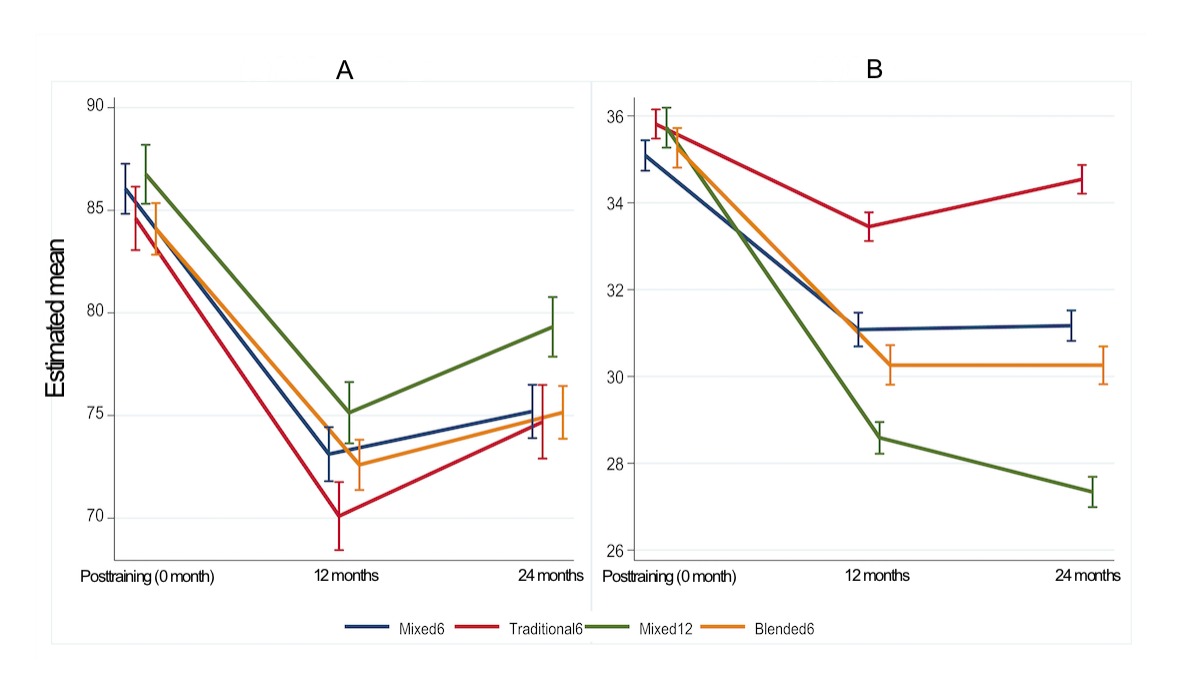
Estimated mean scores with 95% CI for (A) BLS knowledge and (B) skill tests in different training courses by generalized estimating equation models. BLS: basic life support.

Multimedia Appendix 7 provides the descriptive statistics for the posttraining follow-up data. The results revealed that the Mixed12 group exhibited consistent BLS knowledge scores at baseline (postinitial training), with the highest average scores observed at 12 and 24 months after training. The Traditional6 group exhibited the highest average scores on the skill test at all 3 measurement time points. Figure 3 illustrates the estimated mean scores of BLS knowledge and skill tests for each group, as assessed over time using GEE models. At 12 months after initial training, the Traditional6 group had the lowest average BLS knowledge score (mean 70.10, SE 0.854), which was significantly different from that of the Mixed12 group (mean 75.14, SE 0.762; Figure 3A presents a nonoverlapping 95% CI). Subsequently, at 24 months following initial training, the Mixed12 group exhibited significantly higher scores (mean 79.32, SE 0.741) compared with the other groups.

Furthermore, at baseline, a notable difference was observed in the average scores of the skill tests between the Mixed6 and Traditional6 groups (*P*=.003; Figure 3B shows a nonoverlapping 95% CI). Moreover, in the follow-up assessment, the Traditional6 group exhibited significantly higher scores than the other groups. Table 3 presents the proportion in each group for the achievement of high-quality CPR. At 12 and 24 months after initial training, this proportion in the Mixed12 group exhibited the most substantial decrease compared with those at 12 and 24 months after training. At baseline, no substantial differences were observed in these proportions among the 4 groups. However, no substantial differences were observed among these proportions among the Blend6, Mixed6, and Traditional6 groups at 12 or 24 months after initial training. We concurrently used multiple linear regression and GEE models to examine the performance indicators; the corresponding results are provided in Multimedia Appendices 5, 6, 8, and 9.

**Table 3:** Proportions of the achievement of high-quality CPRa at 0, 12, and 24 months after training for the different training courses.

Variables	Mixed6 (n=332), n (%)	Traditional6(n=270), n (%)	Mixed12 (n=258), n (%)	Blended6 (n=303), n (%)
Posttraining (0 month)	91 (27.4)	86 (31.9)	79 (30.6)	98 (32.3)
Posttraining (12 months)	83 (25)	61 (22.6)	2 (0.8)	63 (20.8)
Posttraining (24 months)	79 (23.8)	53 (19.6)	7 (2.7)	84 (27.7)

^a^CPR: cardiopulmonary resuscitation.

**Figure 4:**
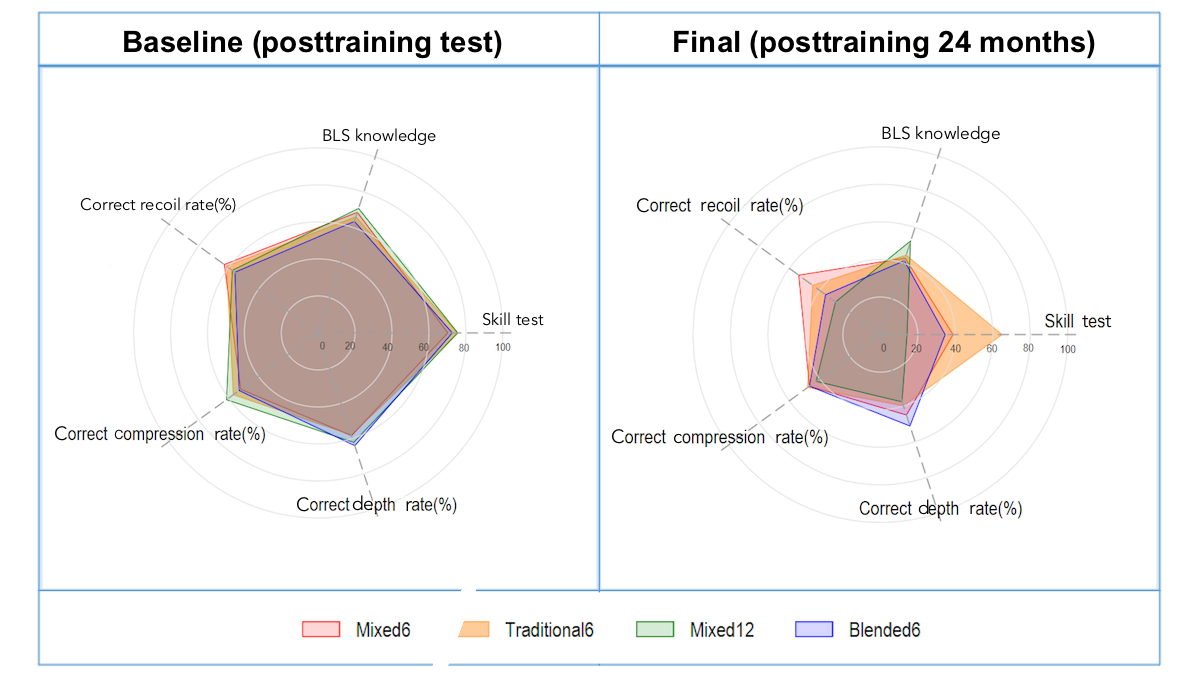
Radar charts for posttraining evaluation at baseline and final visit (posttraining 24 months). BLS: basic life support.

We used an alternative method to rank the 4 training methods based on objectively evaluated items. The scores were converted using percent ranking, and the average score was then calculated to represent the performance of each skill in each training method. Subsequently, we visualized the results as a radar chart (Figure 4). Overall, the 4 groups exhibited comparable average performance in the tests after the first training session. However, in the follow-up assessment (ie, 12 and 24 months after training), differences emerged among the groups (Multimedia Appendix 10). The Traditional6 group exhibited outstanding performance in the skill test and correct recoil rate. The Blended6 group demonstrated superiority in correct depth rate, whereas no significant difference was observed between the Blended6 and Traditional6 groups in terms of correct compression rate or high-quality CPR achievement. The Mixed12 group exhibited a lower correct recoil rate, compression rate, depth rate, and skill test performance compared with the other 3 groups.

## Discussion

### Principal Findings

This study provides 3 major findings regarding the effectiveness of traditional and blended training methods for CPR education. First, no significant difference was observed in knowledge acquisition after initial training, and all the training groups exhibited proficient CPR skills that met the requirements for high-quality CPR. However, a higher proportion of participants receiving blended training initially achieved high-quality CPR; this finding served as the basis for our comparative analysis. The second major finding highlights the importance of timely retraining. When retraining was conducted 12 months after initial training, significant decreases were observed in the proficiency of CPR skills and the proportion of participants achieving high-quality CPR. Our third major finding suggests that more frequent retraining could maintain CPR skills more effectively. The participants who underwent retraining every 6 months exhibited slight decreases in their proficiency in CPR skills and their achievement of high-quality CPR. Additionally, we explored the potential of web-based self-directed learning as an alternative, and this learning method demonstrated effectiveness for skill retention regardless of the initial training method (traditional or blended), with no significant difference observed between the 2 methods.

Research has demonstrated that blended learning and traditional CPR methods [19,21,22] are practical and reasonably effective alternatives to traditional CPR training; however, large-scale comparisons of these methods or the integration of these instructional methods into CPR education have not been conducted. To the best of our knowledge, this study was the first study to demonstrate that blended learning and retraining stimuli are not inferior to traditional methods when it comes to CPR performance. Chien et al [15] found that blended learning for CPR training does not have inferior learning outcomes relative to traditional methods but that CPR skills at 6 months did not meet the AHA’s CPR guidelines. This finding was consistent with our findings. Although traditional instruction may lead to slightly more favorable performance initially, providing self-directed blended learning stimuli every 6 months is effective for maintaining CPR skills. We found that among learners who received CPR training every 12 months, the performance of high-quality CPR decreased by 35% more than that of those retrained every 6 months. Therefore, consistent with previous research recommendations, stimulating learning every 6 months appears to be favorable to doing so every 12 months. This observation aligns with the AHA’s 2020 guidelines, which suggest that for the general public, the use of convenient learning methods alongside retraining is a viable alternative to traditional face-to-face CPR training.

The blended learning method used in this study offers considerable economic benefits and is time saving for both learners and instructors. By incorporating 18 minutes of web-based learning and self-training into a course, the face-to-face instruction and relearning time were collectively shortened by approximately 72 minutes initially and by 12 minutes in subsequent training. These decreases reduced the expenditure, human resources, and time requirements for learners and instructors in CPR training courses [21]. One study investigated the cost-effectiveness of blended learning for CPR training; the results revealed that blended learning decreased training costs while achieving similar maintenance of CPR skills relative to the traditional method [23]. However, some researchers have indicated that despite the costs and time reductions offered by blended learning, such learning does not ensure that participants will acquire further professional knowledge and proficiency in a demanding training environment [22]. The maintenance of CPR skills contributes to the willingness of the public to perform CPR. When EMSs are activated, guiding individuals to identify cardiac arrest and to implement CPR with dispatcher assistance is challenging as is ensuring that members of the public are able to perform high-quality CPR [24]. Accordingly, blended teaching and retraining models, which appear to be as effective as traditional learning models, can address the challenge of instructing individuals during emergency calls. The characteristics of blended teaching models, including time saving and environmental efficiency, can be beneficial for promoting CPR education among the public and for addressing challenges in maintaining CPR skills among the public.

In this study, 95.1%% (1106/1163) of the participants were high school graduates who were approximately 40 years old and who exhibited higher learning and web-based operating abilities. This demographic advantage likely contributed to the success of blended learning in this study. Moreover, this study used a participant-to-manikin ratio of 2-3:1, leading to higher costs compared with the traditional method (1 manikin to 6 students). The increased investment in training infrastructure may affect the overall cost-effectiveness of blended learning in various settings. The study did not record the frequency of learners’ usage of blended relearning stimuli; the effectiveness of self-paced web-based learning may be related to the time spent engaging with the material. Nevertheless, the primary objective of blended web-based learning is to enable individuals to learn at their convenience. In contrast to traditional face-to-face classroom learning, in blended learning, participants have the flexibility to arrange their web-based and in-class training according to their convenience and location. Accordingly, this learner-centric approach can lead to an environment that is more conducive to the maintenance of CPR skills.

In this study, favorable exercise habits and previous CPR learning experiences enhanced the effectiveness of CPR training. Even if learning had occurred more than 2 years previously, blended CPR training could effectively maintain CPR skills. Ettl et al [20] found that incorporating CPR learning into fitness exercise training increased learners’ motivation and confidence in performing CPR. Therefore, establishing exercise habits helps maintain CPR skills and for fostering rescue skills.

Finally, although blended learning with a retraining frequency of 6 months demonstrated significant economic benefits and time-saving ability in this study, its cost-effectiveness depended on factors such as participant demographics, the training environment, and the level of engagement with web-based learning opportunities. Accordingly, consideration of these factors could maximize the potential of blended learning in various CPR training scenarios.

### Limitations

This study had some limitations. First, in observational studies, the random allocation of samples is infeasible and could result in disparities between groups. Consequently, we used a multivariate regression model to mitigate the impact of variables; thus, we impartially assessed the differences between the groups. Moreover, this study involved tracking the training status of each group to understand the importance of the interval between retraining sessions and whether the given training method was appropriate. Second, we collected demographic data from a subset of learners, but our comprehension of these learners’ economic backgrounds and technology use was limited; consequently, whether blended learning is effective among individuals with relatively low socioeconomic status should be further explored. Third, our research cohort lacked the representation of older adults. As a result, uncertainties persist regarding the applicability of blended training for this demographic; accordingly, future studies are recommended to address this crucial gap. Finally, the absence of an analysis of the participants’ willingness to perform CPR leaves a significant gap in our understanding. Accordingly, individuals’ willingness to administer CPR after blended retraining should be investigated in future research.

### Conclusions

Blended learning for CPR with a retraining frequency of 6 months provides higher retention of high-quality CPR skills than does retraining every 12 months. Notably, the blended method demonstrated effects similar to those of traditional relearning methods.

## Appendix

Multimedia Appendix 1 The written test of cardiopulmonary resuscitation knowledge.

Multimedia Appendix 2 The skill test of cardiopulmonary resuscitation practice checklist.

Multimedia Appendix 3 Generalized estimating equation models for the performance indicators.

Multimedia Appendix 4 Generalized estimating equation models for the performance indicators.

Multimedia Appendix 5 Multiple linear regression model for the performance indicators at baseline: basic life support knowledge, skill test, average compression depth, and rate.

Multimedia Appendix 6 Multiple analysis for the performance indicators at baseline: the proportion of correct compression depth, speed rate, and recoil.

Multimedia Appendix 7 Summary statistics for outcome assessment at baseline, post-12M, post- 24M in different training courses.

Multimedia Appendix 8 Estimated mean with 95% CI for compression depth and rate in different training courses by generalized estimating equation models.

Multimedia Appendix 9 Estimated mean with 95% CI for correct compression depth, rate, and recoil in different training courses by generalized estimating equation models.

Multimedia Appendix 10 The radar chart for posttraining evaluation after 12 months.

## Abbreviations

AED: automated external defibrillator
AHA: American Heart Association
BLS: basic life support
bpm: beats per minute
CPR: cardiopulmonary resuscitation
EMS: emergency medical service
GEE: generalized estimating equation
OHCA: out-of-hospital cardiac arrest

## Data Availability

The data sets generated or analyzed during this study are available from the corresponding author upon reasonable request.
