# Supplementary material for: Effectiveness of Blended Versus Traditional Refresher Training for Cardiopulmonary Resuscitation: Prospective Observational Study
Source: JMIR Med Educ. 2024 Apr 29;10:e52230. doi: 10.2196/52230 (PMC11091803; doi:10.2196/52230)
Supplement: Multimedia Appendix 3 [file mededu_v10i1e52230_app3.docx]

**Multimedia Appendix 3.** GEE models for the performance indicators.

|  | BLS knowledge  aβ(95% CI) | Skill test  aβ(95% CI) | Avg. compression depth (cm)  aβ(95% CI) | Avg. compression rate (times/ min)  aβ(95% CI) |
| --- | --- | --- | --- | --- |
| Intercept | 86.05 (84.83, 87.27) | 35.81 (35.48, 36.15) | 5.01 (4.93, 5.10) | 110.56 (108.86, 112.27) |
| Mixed6 | −1.44 (−3.41, 0.53) | −0.72 (−1.20, −0.24) | 0.05 (−0.06, 0.17) | 3.32 (1.05, 5.59) |
| Traditional6 | ref | ref | ref | ref |
| Mixed12 | 0.7 (−1.18, 2.59) | −0.08 (−0.65, 0.48) | 0.22 (0.12, 0.32) | 5.51 (3.32, 7.71) |
| Blended6 | −1.96 (−3.71, −0.20) | −0.55 (−1.12, 0.01) | 0.32 (0.21, 0.43) | 6.09 (4.03, 8.15) |
| Baseline | ref | ref | ref | ref |
| Post 12M | −12.93 (−13.68, −12.19) | −2.37 (−2.42, −2.31) | −0.26 (−0.29, −0.23) | −4.00 (−4.00, −4.00) |
| Post 12M*Mixed6 | −1.57 (−2.61, −0.53) | −1.65 (−1.76, −1.54) | 0.09 (0.04, 0.13) | 6.33 (5.51, 7.16) |
| Post 12M*Mixed12 | 1.31 (0.47, 2.16) | −4.78 (−4.89, −4.67) | −0.37 (−0.40, −0.34) | 13.8 (13.73, 13.87) |
| Post 12M*Blended6 | 1.43 (0.63, 2.24) | −2.63 (−2.69, −2.58) | −0.19 (−0.22, −0.16) | 3.68 (3.12, 4.25) |
| Post 24M | −10.85 (−11.54, −10.16) | −1.28 (−1.32, −1.23) | −0.33 (−0.36, −0.30) | 0.01 (−0.17, 0.19) |
| Post 24M*Mixed6 | 0.94 (−0.23, 2.10) | −2.65 (−2.77, −2.52) | 0.05 (−0.02, 0.11) | 1.57 (1.12, 2.02) |
| Post 24M*Mixed12 | 3.41 (2.65, 4.18) | −7.12 (−7.24, −7.01) | −0.21 (−0.24, −0.18) | 7.2 (7.01, 7.38) |
| Post 24M*Blended6 | 1.91 (1.05, 2.77) | −3.73 (−3.78, −3.67) | 0.03 (−0.0003, 0.07) | −1.74 (−2.17, −1.31) |

aβ: the estimate of the parameter adjusted by age, gender, education, Exercise habits, first time for CPR training and pre BLS knowledge score.
