# Supplementary material for: Effectiveness of Blended Versus Traditional Refresher Training for Cardiopulmonary Resuscitation: Prospective Observational Study
Source: JMIR Med Educ. 2024 Apr 29;10:e52230. doi: 10.2196/52230 (PMC11091803; doi:10.2196/52230)
Supplement: Multimedia Appendix 4 [file mededu_v10i1e52230_app4.docx]

**Multimedia Appendix 4.** GEE models for the performance indicators.

|  | Correct compression depth (%)  aβ(95% CI) | Correct compression rate (%)  aβ(95% CI) | Correct recoil (%)  aβ(95% CI) | High quality CPR achievement†  aβ(95% CI) |
| --- | --- | --- | --- | --- |
| *Intercept* | *71.24 (67.60, 74.88)* | *66.16 (62.52, 69.80)* | *77.16 (73.55, 80.77)* | *−0.76 (−1.02, −0.50)* |
| Mixed6 | −0.45 (−5.52, 4.61) | **−5.03 (−10.02, −0.03)** | −2.77 (−8.01, 2.46) | −0.21 (−0.57, 0.14) |
| Traditional6 | ref | ref | ref | ref |
| Mixed12 | 3.51 (−1.84, 8.86) | 2.45 (−3.08, 7.97) | **−7.44 (−13.27, −1.61)** | **−0.43 (−0.79, −0.08)** |
| Blended6 | 4.64 (−0.58, 9.86) | −5.18 (−10.54, 0.17) | **−6.81 (−12.20, −1.42)** | 0.28 (−0.06, 0.63) |
| Baseline | ref | ref | ref |  |
| Post 12M | **−13.11 (−15.00, −11.22)** | −1.48 (−6.58, 3.62) | **−2.02 (−3.50, −0.55)** | **−0.47 (−0.66, −0.28)** |
| Post 12M*Mixed6 | **3.3 (1.01, 5.58)** | −4.45 (−9.56, 0.66) | 7.76 (4.63, 10.89) | **0.35 (0.02, 0.67)** |
| Post 12M*Mixed12 | 1.7 (−0.28, 3.69) | **−9.5 (−14.64, −4.36)** | −8.44 (−10.06, −6.81) | **−4.05 (−5.48, −2.63)** |
| Post 12M*Blended6 | **5.52 (3.59, 7.45)** | −2.79 (−7.90, 2.32) | −3.61 (−5.11, −2.10) | **0.49 (0.18, 0.80)** |
| Post 24M | **−14.71 (−17.53, −11.89)** | **−3.63 (−6.62, −0.64)** | **−4.21 (−5.96, −2.45)** | **−0.65 (−0.90, −0.40)** |
| Post 24M*Mixed6 | **4.74 (1.64, 7.84)** | −0.33 (−3.33, 2.66) | 7.84 (4.57, 11.11) | **0.46 (0.15, 0.77)** |
| Post 24M*Mixed12 | 1.25 (−1.65, 4.16) | **−10.09 (−13.19, −6.99)** | **−9.04 (−11.00, −7.08)** | **−2.60 (−3.40, −1.81)** |
| Post 24M*Blended6 | **8.39 (5.55, 11.22)** | −0.84 (−3.84, 2.16) | **−4.47 (−6.30, −2.64)** | 0.17 (−0.14, 0.48) |

aβ: the estimate of the parameter adjusted by age, gender, education, Exercise habits, first time for CPR training and pre BLS knowledge score.

† The GEE model was constructed with the logit link; the others with the identity link. Odds ratio can be transformed by Exp(β).

Statistical significances are marked in bold
