# Supplementary material for: Effectiveness of Blended Versus Traditional Refresher Training for Cardiopulmonary Resuscitation: Prospective Observational Study
Source: JMIR Med Educ. 2024 Apr 29;10:e52230. doi: 10.2196/52230 (PMC11091803; doi:10.2196/52230)
Supplement: Multimedia Appendix 5 [file mededu_v10i1e52230_app5.docx]

**Multimedia Appendix 5.** Multiple linear regression model for the performance indicators at baseline: BLS knowledge, skill test, average compression depth and rate.

| Outcome measurement: | BLS knowledge | | Skill test | | Avg. compression depth (cm) | | Avg. compression rate (times/ min) | |
| --- | --- | --- | --- | --- | --- | --- | --- | --- |
| Covariates | aβ(95% CI) | p-value | aβ(95% CI) | p-value | aβ(95% CI) | p-value | aβ(95% CI) | p-value |
| Group |  |  |  | |  |  |  | |
| Mixed6 | 1.44 (−0.45, 3.34) | 0.135 | −0.72 (−1.28, −0.16) | **0.012** | 0.05 (−0.05, 0.16) | 0.302 | 3.32 (1.30, 5.34) | **0.001** |
| Traditional6 (baseline) | Ref |  | Ref |  | ref |  | ref |  |
| Mixed12 | 2.15 (0.14, 4.16) | **0.036** | −0.08 (−0.68, 0.51) | 0.787 | 0.22 (0.11, 0.33) | **<0.001** | 5.51 (3.36, 7.66) | **<0.001** |
| Blended6 | −0.51 (−2.45, 1.42) | 0.604 | −0.55 (−1.13, 0.02) | 0.058 | 0.32 (0.22, 0.42) | **<0.001** | 6.09 (4.03, 8.15) | **<0.001** |
| Age | −0.06 (−0.12, 0.00) | **0.044** | −0.03 (−0.04, −0.01) | **0.003** | −0.004 (−0.007, 0.001) | **0.015** | −0.06 (−0.126, 0.001) | **0.053** |
| Gender (M vs. F) | 1.28 (−0.13, 2.69) | 0.074 | 0.17 (−0.25, 0.59) | 0.416 | −0.13 (−0.21, −0.05) | **<0.001** | 0.71 (−0.82, 2.24) | 0.364 |
| Education (higher vs. low) | 9.51 (5.75, 13.27) | **<0.001** | 2.31 (1.19, 3.43) | **<0.001** | 0.20 (−0.01, 0.41) | 0.058 | 4.37 (0.30, 8.44) | **0.035** |
| Exercise habits (Y vs. N) | 0.04 (−1.36, 1.43) | 0.959 | 0.22 (−0.19, 0.63) | 0.285 | 0.01 (−0.07, 0.08) | 0.895 | 0.47 (−1.03, 1.97) | 0.538 |
| First time for CPR training (have ever had vs. the first-time) | 3.23 1.54, 4.92) | **<0.001** | 0.54 (0.04, 1.04) | **0.035** | 0.08 (−0.01, 0.17) | 0.085 | 2.27 (−0.44, 4.10) | **0.015** |
| Pre BLS knowledge score | 0.25 (0.21, 0.29) | **<0.001** | 0.02 (0.01, 0.03) | **0.004** | 0.001 (−0.001, 0.004) | 0.344 | 0.040(−0.01, 0.09) | 0.107 |

aβ: the estimate of the parameter adjusted by age, gender, education, Exercise habits, first time for CPR training and pre BLS knowledge score.
