# Supplementary material for: Effectiveness of Blended Versus Traditional Refresher Training for Cardiopulmonary Resuscitation: Prospective Observational Study
Source: JMIR Med Educ. 2024 Apr 29;10:e52230. doi: 10.2196/52230 (PMC11091803; doi:10.2196/52230)
Supplement: Multimedia Appendix 6 [file mededu_v10i1e52230_app6.docx]

**Multimedia Appendix 6.** Multiple analysis for the performance indicators at baseline: the proportion of correct compression depth, speed rate and recoil.

| Outcome measurement: | Correct compression depth (%) | | Correct compression rate (%) | | Correct recoil (%) | | High quality CPR achievement | |
| --- | --- | --- | --- | --- | --- | --- | --- | --- |
| Covariates | aβ(95% CI) | p-value | aβ(95% CI) | p-value | aβ(95% CI) | p-value | aOR (95% CI) | p-value |
| Group |  |  |  |  |  |  |  |  |
| Mixed6 | −0.45 (−5.63, 4.73) | 0.864 | −5.03 (−10.31, 0.25) | 0.062 | −2.77 (−8.36, 2.81) | 0.330 | 1.24 (0.87, 1.76) | 0.234 |
| Traditional6 (baseline) | ref |  | ref |  | ref |  | Ref |  |
| Mixed12 | 3.51 (−2.00, 9.01) | 0.212 | 2.45 (−3.16, 8.05) | 0.393 | −7.44 (−13.38, −1.51) | 0.014 | 0.65 (0.45, 0.93) | 0.017 |
| Blended6 | 4.64 (−0.65, 9.93) | 0.086 | 5.18 (−0.21, 10.57) | 0.059 | −6.81 (−12.51, −1.11) | 0.019 | 1.15 (0.82, 1.57) | 0.108 |
| Age | −0.16 (−0.32, 0.003) | 0.055 | −0.11 (−0.28, 0.05) | 0.180 | −0.005 (−0.18, 0.17) | 0.957 | 1.01 (1.002, 1.02) | 0.019 |
| Gender (Male vs. Female) | −3.35 (−7.21, 0.52) | 0.090 | −2.28 (−6.23, 1.66) | 0.256 | 11.86 (7.75, 15.97) | <0.001 | 0.96 (0.74, 1.23) | 0.729 |
| Education (higher vs. low) | 7.89 (−2.42, 18.20) | 0.133 | −3.13 (−13.66, 7.40) | 0.561 | −9.40 (−20.53, 1.72) | 0.098 | 0.84 (0.42, 1.68) | 0.627 |
| Exercise habits (Yes vs. No) | −1.89 (−5.70, 1.91) | 0.330 | −2.55 (−6.43, 1.34) | 0.198 | −6.06 (−10.16, −1.96) | 0.004 | 1.20 (0.94, 1.54) | 0.148 |
| First time for CPR training (first-time vs have ever had) | −5.21 (−9.84, −0.57) | 0.028 | 0.74 (−3.99, 5.46) | 0.760 | 3.45 (−1.56, 8.45) | 0.177 | 1.09 (0.81, 1.48) | 0.566 |
| Pre BLS knowledge score | −0.04 (−0.16, 0.09) | 0.540 | −0.006 (−0.13, 0.12) | 0.931 | −0.063 (−0.196, 0.070) | 0.355 | 1.00 (0.99, 1.01) | 0.967 |

aβ: the estimate of the parameter adjusted by age, gender, education, Exercise habits, first time for CPR training and pre BLS knowledge score.

aOR: odds ratio adjusted by age, gender, education, Exercise habits, first time for CPR training and pre BLS knowledge score.
