# Supplementary material for: Effectiveness of Blended Versus Traditional Refresher Training for Cardiopulmonary Resuscitation: Prospective Observational Study
Source: JMIR Med Educ. 2024 Apr 29;10:e52230. doi: 10.2196/52230 (PMC11091803; doi:10.2196/52230)
Supplement: Multimedia Appendix 7 [file mededu_v10i1e52230_app7.docx]

**Multimedia Appendix 7.** Summary statistics for outcome assessment at baseline, post-12M, post-24M in different training courses.

| Mean ± SD | | Mixed6 | Traditional6 | | Mixed12 | | Blended6 | |
| --- | --- | --- | --- | --- | --- | --- | --- | --- |
| BLS knowledge score | |  |  | |  | |  | |
| post-training(0M) | | 86.05 ± 11.38 | 84.61 ± 12.96 | | 86.76 ± 11.79 | | 84.10 ± 11.19 | |
| 12M | | 73.12 ± 12.27 | 70.10 ± 13.92 | | 75.14 ± 12.27 | | 72.60 ± 10.90 | |
| 24M | | 75.20 ± 12.11 | 74.69 ± 15.05 | | 79.32 ± 11.93 | | 75.15 ± 11.46 | |
| Skill test score | |  |  | |  | |  | |
| post-training(0M) | | 35.09 ± 3.26 | 35.81 ± 2.78 | | 35.73 ± 3.76 | | 35.26 ± 4.05 | |
| 12M | | 31.08 ± 3.63 | 33.45 ± 2.77 | | 28.59 ± 3.01 | | 30.26 ± 4.05 | |
| 24M | | 31.17 ± 3.24 | 34.54 ± 2.74 | | 27.34 ± 2.88 | | 30.26 ± 3.89 | |
| Average chest compression depth, cm |  | |  | |  | |  | |
| post-training(0M) | | 5.07 ± 0.74 | 5.01 ± 0.73 | | 5.23 ± 0.43 | | 5.33 ± 0.57 | |
| 12M | | 4.89 ± 0.72 | 4.75 ± 0.68 | | 4.60 ± 0.37 | | 4.88 ± 0.53 | |
| 24M | | 4.78 ± 0.79 | 4.68 ± 0.69 | | 4.69 ± 0.37 | | 5.04 ± 0.58 | |
| Average chest compression rate, times/min | |  |  | |  | |  | |
| post-training(0M) | | 113.88 ± 13.87 | 110.56 ± 14.34 | | 116.07 ± 11.33 | | 116.65 ± 10.28 | |
| 12M | | 116.21 ± 15.42 | 106.56 ± 14.34 | | 125.88 ± 11.90 | | 116.34 ± 11.69 | |
| 24M | | 115.46 ± 14.05 | 110.57 ± 14.23 | | 123.28 ± 11.71 | | 114.92 ± 10.87 | |
| High quality CPR | | | |  | |  | |  |
| post-training(0M) | | 91 (27.4) | | 86 (31.9) | | 77 (29.8) | | 98 (32.3) |
| 12M | | 83 (25.0) | | 61 (22.6) | | 2 (0.8) | | 64 (21.1) |
| 24M | | 79 (23.8) | | 53 (19.6) | | 7 (2.7) | | 84 (27.7) |
