# Supplementary material for: Effectiveness of Blended Versus Traditional Refresher Training for Cardiopulmonary Resuscitation: Prospective Observational Study
Source: JMIR Med Educ. 2024 Apr 29;10:e52230. doi: 10.2196/52230 (PMC11091803; doi:10.2196/52230)
Supplement: Multimedia Appendix 8 [file mededu_v10i1e52230_app8.pdf]

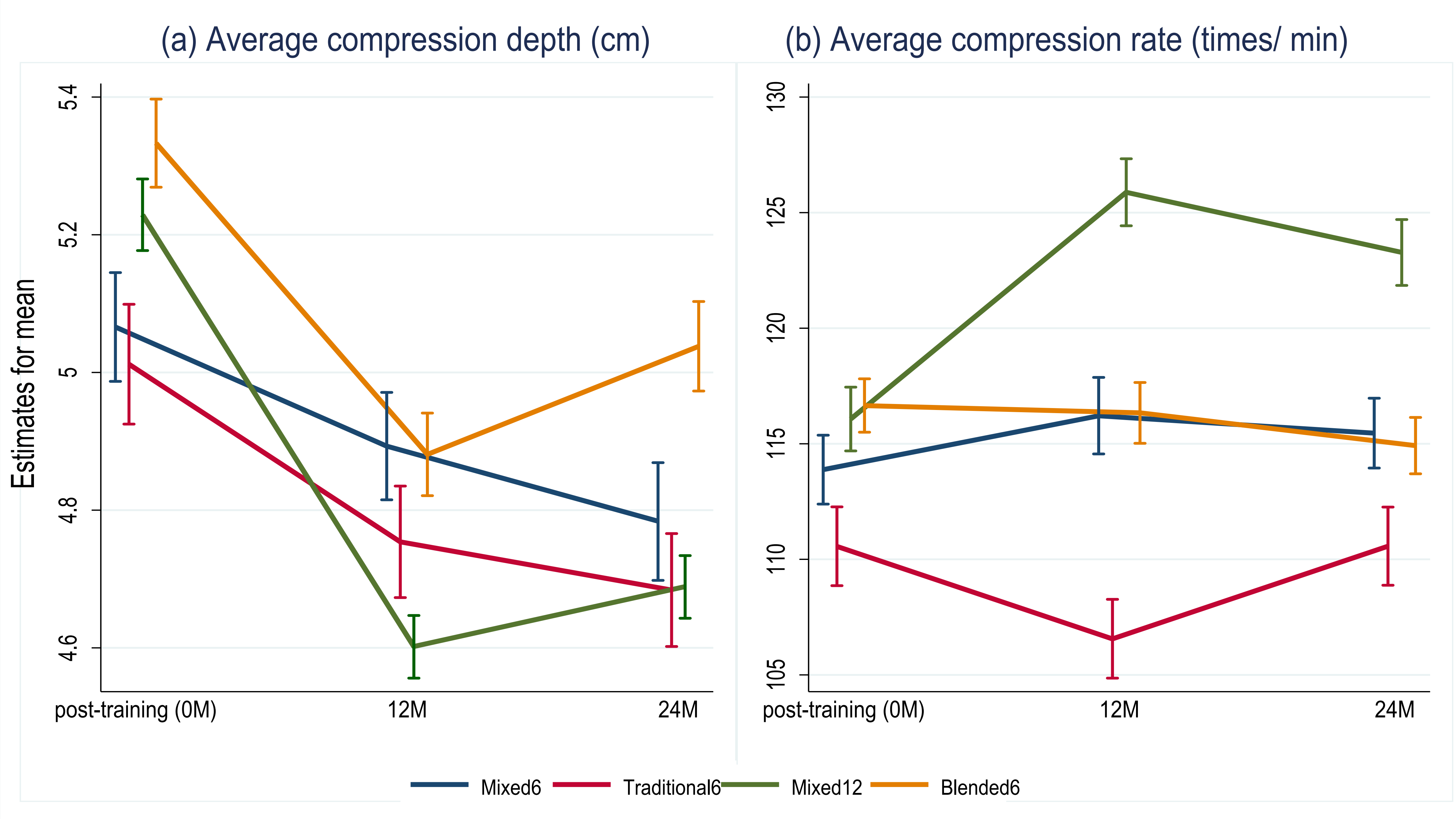

(a) Average compression depth (cm)
(b) Average compression rate (times/ min)
Estimates for mean
5.4
5.2
5
4.8
4.6
130
125
120
115
110
105
post-training (0M)
12M
24M
post-training (0M)
12M
24M
Mixed6
Traditional6
Mixed12
Blended6
