# Supplementary figures and images for: Effectiveness of Blended Versus Traditional Refresher Training for Cardiopulmonary Resuscitation: Prospective Observational Study
Source: JMIR Med Educ. 2024 Apr 29;10:e52230. doi: 10.2196/52230 (PMC11091803; doi:10.2196/52230)

## Achievement of CPR

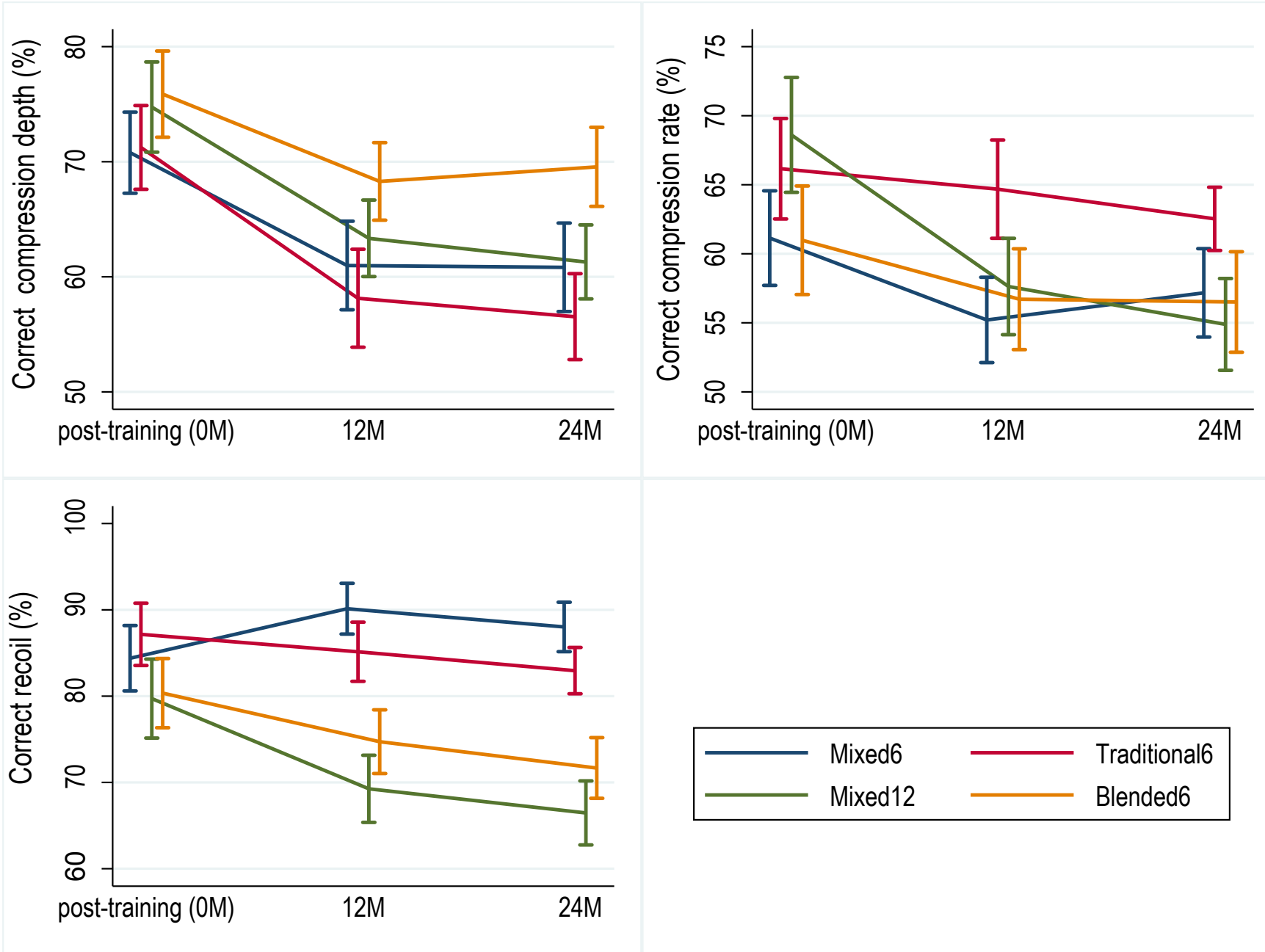

Supplement: Multimedia Appendix 9 [file mededu_v10i1e52230_app9.pdf]

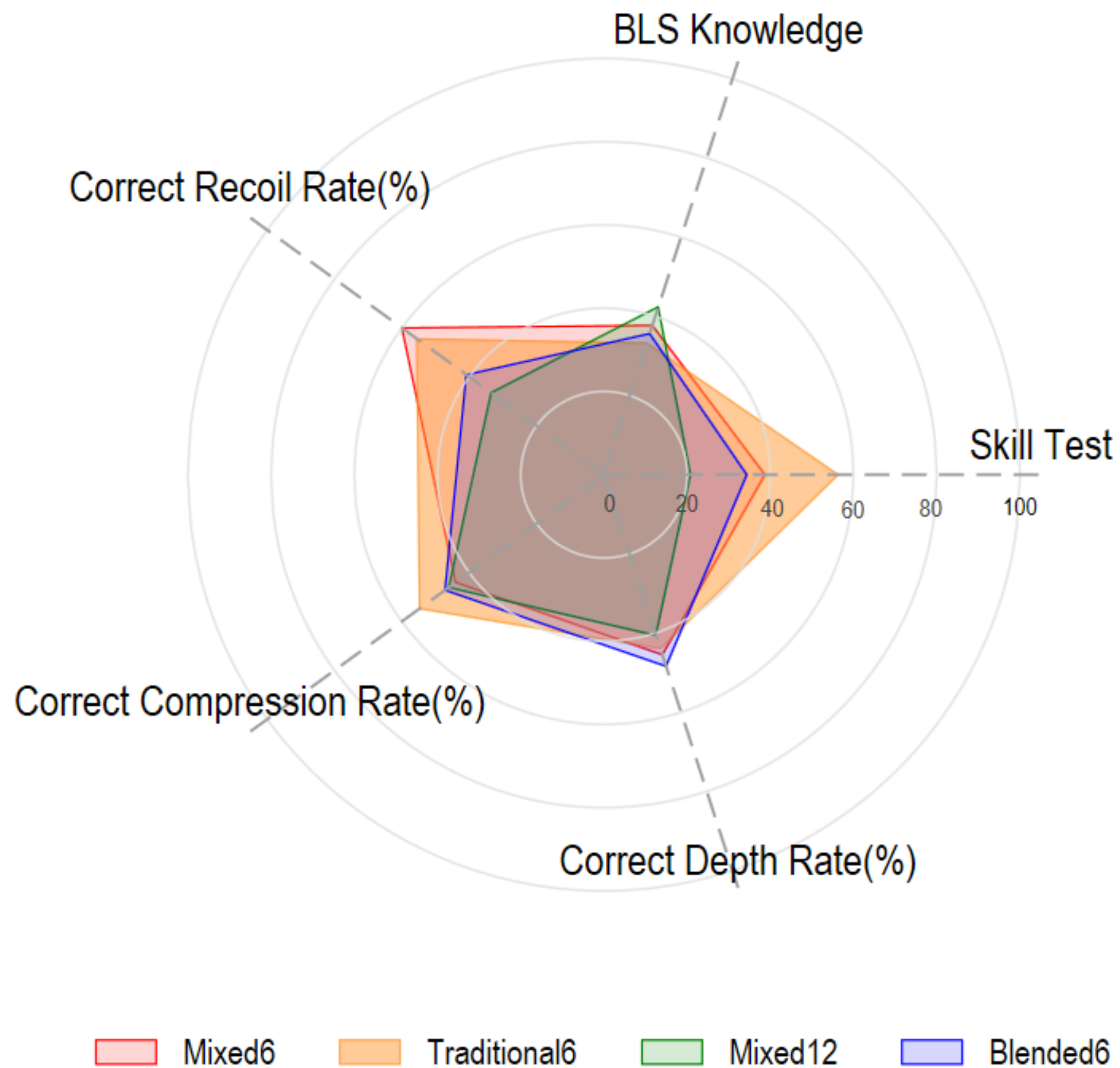

Supplement: Multimedia Appendix 10 [file mededu_v10i1e52230_app10.pdf]
